# Ventral striatal dopamine synthesis capacity is associated with individual differences in behavioral disinhibition

**DOI:** 10.3389/fnbeh.2014.00086

**Published:** 2014-03-14

**Authors:** Andrew D. Lawrence, David J. Brooks

**Affiliations:** ^1^School of Psychology, Cardiff UniversityCardiff, UK; ^2^Division of Brain Sciences, Department of Medicine, Imperial CollegeLondon, UK; ^3^Department of Nuclear Medicine, PET Centre, Aarhus UniversityAarhus, Denmark

**Keywords:** addiction, dopamine, externalizing, impulsivity, positron emission tomography, pathological gambling, reward, ventral striatum

## Abstract

Pathological gambling, alongside addictive and antisocial disorders, forms part of a broad psychopathological spectrum of externalizing disorders, which share an underlying genetic vulnerability. The shared externalizing propensity is a highly heritable, continuously varying trait. Disinhibitory personality traits such as impulsivity and novelty seeking (NS) function as indicators of this broad shared externalizing tendency, which may reflect, at the neurobiological level, variation in the reactivity of dopaminergic (DAergic) brain reward systems centered on the ventral striatum (VS). Here, we examined whether individual differences in ventral striatal dopamine (DA) synthesis capacity were associated with individual variation in disinhibitory personality traits. Twelve healthy male volunteers underwent 6-[^18^F]Fluoro-L-DOPA (FDOPA) positron emission tomography (PET) scanning to measure striatal DA synthesis capacity, and completed a measure of disinhibited personality (NS). We found that levels of ventral, but not dorsal, striatal DA synthesis capacity were significantly correlated with inter-individual variation in disinhibitory personality traits, particularly a propensity for financial extravagance and irresponsibility. Our results are consistent with preclinical models of behavioral disinhibition and addiction proneness, and provide novel insights into the neurobiology of personality based vulnerability to pathological gambling and other externalizing disorders.

## Introduction

Patterns of systematic co-occurrence (“comorbidity”) between substance misuse and antisocial disorders are best accounted for by a model positing a shared underlying genetic vulnerability, known as externalizing (Krueger et al., 2002, 2007). This broad externalizing vulnerability is a highly heritable, continuously varying dimension of risk (Krueger et al., 2007). Pathological gambling [now called gambling disorder] systematically co-occurs with both substance misuse and antisocial disorders (Kessler et al., 2008; Oleski et al., 2011) and this co-variation likewise reflects a shared genetic vulnerability (Slutske et al., 2001, 2013; Blanco et al., 2012). Thus, pathological gambling can be considered one variant of an externalizing spectrum of disorders.

The broad personality trait of disinhibition reflects individual differences in the tendency to behave in a disinhibited vs. controlled fashion (Dindo et al., 2009). Disinhibitory personality traits are strongly linked with externalizing disorders (Ruiz et al., 2008), including pathological gambling (MacLaren et al., 2011). Importantly, a shared genetic diathesis underlies the associations between trait disinhibition and externalizing disorders (Krueger et al., 2002; Hicks et al., 2011).

Furthermore, prospective studies suggest that trait disinhibition, measured early in life, predates and predicts the emergence of externalizing pathology, including pathological gambling (Elkins et al., 2006; Slutske et al., 2012) and mediates the co-variation between externalizing disorders (Ruiz et al., 2008). Thus the antecedent trait of disinhibition provides the temperamental core of the externalizing disorders, and disinhibitory personality traits such as impulsivity and sensation seeking function as indicators of the general externalizing propensity (Krueger et al., 2002, 2007).

The genetic liability to externalizing may be related, at the neurobiological level, to brain mechanisms underpinning sensitivity to reward (Iacono et al., 2008). Brain dopamine (DA) systems have long been hypothesized to underlie individual variation in reward sensitivity. According to Gray (1987), individual differences in trait impulsivity reflect individual variation in the reactivity of a neural “behavioral activation system” (BAS), centred on the ventral striatum (VS) and its dopaminergic (DAergic) irrigation, which is triggered by cues for reward. Likewise, in Cloninger’s (1986) model of temperament, novelty-seeking (NS) tendencies reflect genetically determined variation in reward-seeking behaviors, mediated by DAergic modulation of the BAS. When activated, the BAS functions as an impulsive “go” motivational system, and variation in BAS reactivity is potentially a potent source of inter-individual variation in behavioral disinhibition (Newman and Wallace, 1993).

Recent research highlights that genetic variation in DA synthesis pathways may play a key role in the etiology of externalizing liability. DA synthesis occurs within DA neurons. Tyrosine is transported into the cell via amino acid carriers in the blood-brain barrier and cell membranes. Once in the intracellular space it is hydroxylated to L-3,4-dihydroxiphenylalanine (L-DOPA) by tyrosine hydroxylase (TH). L-DOPA is then decarboxylated by aromatic L-amino acid decarboxylase (AADC) (also called dopa decarboxylase, DDC) to DA (Elsworth and Roth, 2009). In an important study, Derringer et al. (2010) found that a combination of multiple common variants (single nucleotide polymorphisms, SNPs) in the DDC gene predicted individual variation in sensation seeking traits, suggesting that genetic variation in DA synthesis contributes to the broad externalizing liability, of which sensation seeking functions as one indicator (Krueger et al., 2007).

Positron Emission Tomography (PET) can be used to study the activity of AADC in pre-synaptic DA terminals in the living brain. The PET tracer 6-[^18^F]fluoro-L-DOPA (FDOPA), a radioactive analog of L-DOPA, the precursor of DA, is taken up by pre-synaptic DAergic neurons and is metabolized by AADC to ^18^F-DA, which is trapped and stored within vesicles in the nerve terminals (Kumakura and Cumming, 2009). FDOPA uptake, quantified as the influx constant *K_i_*, can be used as a measure of AADC activity and vesicular storage capacity (Brown et al., 1999). High values for FDOPA *K_i_* are observed in areas of dense DA nerve terminal innervation, such as the striatum (Kumakura and Cumming, 2009).

Consistent with the notion that externalizing propensity reflects, neuro-biologically, inter-individual variation in DAergic modulation of the BAS, we recently found, in a group of Parkinson’s disease patients, that individual differences in ventral striatal FDOPA *K_i_* values were related to individual differences in disinhibitory personality traits, particularly a propensity for financial extravagance (Lawrence et al., 2013). The patients in that study, were however, being treated with DA agonist medication, which could potentially have influenced levels of both striatal DA synthesis (Rowlett et al., 1993) and behavioral disinhibition (Lawrence et al., 2003). Thus, it is important to ascertain whether the relationship between ventral striatal DA synthesis capacity and disinhibitory personality traits holds in a sample of healthy, medication-free individuals. Based on our previous findings, we predicted that increased FDOPA uptake in ventral, but not dorsal, striatum would be related to increased levels of trait disinhibition, in particular propensities for financial extravagance and irresponsibility.

## Materials and methods

### Participants

Twelve right-handed healthy male volunteers (mean age 38 years, SD ± 7 years, range 29–49 years) participated, all with a normal neurological history and examination. A trained psychiatrist assessed participants and current and past psychiatric morbidity, including alcohol or drug dependency, was excluded by routine psychiatric interview and the General Health Questionnaire (Jackson, 2006) with a cut-off of 5 points or fewer.

The study was limited to men as there are gender differences in the prevalence and clinical presentation of gambling disorder and its relation to the externalizing spectrum (Blanco et al., 2006; Oleski et al., 2011) and in DA synthesis capacity (Laakso et al., 2002). Additionally, fMRI studies suggest a stronger relationship between ventral striatal activity to reward cues and impulsivity in men than women (Lahey et al., 2012).

Permission to undertake the study was granted by the Hammersmith Hospitals Research Ethics Committee and all participants gave written informed consent following a full explanation of the procedure. The Administration of Radioactive Substances Advisory Committee (ARSAC) of the UK approved radioisotope use.

### Personality trait measurement

Our measure of trait behavioral disinhibition was based on NS from Cloninger’s Tri-dimensional Personality Questionnaire (TPQ; Cloninger, 1987). The version of the TPQ used here was a 100-item, self-administered, true-false instrument. The questionnaire is scored so that higher scores reflect greater NS tendencies.

As originally constructed (Cloninger, 1987) TPQ-NS comprised four narrow facet-level scales: Exploratory Excitability vs. Stoic Rigidity (NS1), Impulsiveness vs. Reflection (NS2), Extravagance vs. Reserve (NS3), and Disorderliness vs. Regimentation (NS4). When Ando et al. (2004), however, examined the genetic and environmental factor structure of NS, factor analysis of the genetic inter-correlations yielded factors that did not fully resemble the phenotypic structure of NS as proposed by Cloninger (1987). NS was revised (r-NS) to consist of Impulsiveness vs. Reflection (NS2), Extravagance vs. Reserve (NS3) and Disorderliness vs. Regimentation (NS4), excluding Exploratory Excitability vs. Stoic Rigidity (NS1). Further, Flory and Manuck (2009), using factor analysis in a large normative sample of adults, found Impulsiveness vs. Reflection (NS2) and Extravagance vs. Reserve (NS3) to have high loadings on a “disinhibition” factor, along with the Barratt Impulsiveness Scale (BIS), whereas Exploratory Excitability vs. Stoic Rigidity (NS1) and Disorderliness vs. Regimentation (NS4) loaded on a distinct “Experience seeking” factor.

Hence, in the current study, we focused on those r-NS facets most strongly linked to trait disinhibition: Impulsiveness (vs. Reflection) (NS2) (8 items) and Extravagance (vs. Reserve) (NS3) (7 items). Sample items include “*I often follow my instincts, hunches, or intuition without thinking through all the details”* (Impulsivity, NS2) and “*I often spend money until I run out of cash or get into debt from using too much credit”* (Extravagance, NS3).

In addition to NS, we also measured Harm Avoidance (HA) traits using the TPQ. We calculated a total HA score based on the sum of the four individual HA facet-level scales, as Ando et al. (2004) confirmed Cloninger’s (1987) claim that the subscales used to define HA share a common genetic basis. According to Cloninger (1986), although NS and HA are genetically independent traits, at the phenotypic level high levels of HA should inhibit the expression of NS tendencies, since activation of the HA system results in a “reflexive” or “reactive” form of behavioral inhibition (Carver, 2008)—dampening the expression of appetitive approach behavior and NS, given cues of potential punishment (Newman and Wallace, 1993; Nikolova and Hariri, 2012). Indeed, meta-analysis reveals a consistent strong negative correlation between NS and HA (Miettunen et al., 2008; here the relation between HA and NS3 for example was *r* = −0.44), a relationship that is environmentally (i.e., through experience) and not genetically mediated (Ando et al., 2002). Hence, we controlled for the influence of HA when examining the relation between striatal FDOPA *K_i_* values and NS traits.

### Positron emission tomography (PET) scanning protocol

Participants were pre-treated with 150 mg carbidopa and 400 mg entacapone 1 h prior to radioisotope administration (to block peripheral metabolism of FDOPA and so enhance specific signal detection) and underwent three-dimensional FDOPA PET using an ECAT EXACT HR++ (CTI/Siemens 966) camera, which covers an axial field of view of 23.4 cm and provides 95 transaxial planes. The tomograph has a spatial resolution of 4.8 + 0.2 mm FWHM (transaxial, 1 cm off axis) and 5.6 mm + 0.5 mm (axial, on axis) after image reconstruction (Spinks et al., 2000). A transmission scan, which corrects for attenuation of emitted radiation by skull and tissues, was acquired using a single rotating photon point source of 150 MBq of ^137^Cs. 30 s after the start of the emission scan, 110 (range 102–135) MBq of FDOPA in 10 ml normal saline was infused intravenously over 30 s. Three-dimensional sinograms of emission data were then acquired over 90 min as 26 time frames. Participants were placed in the scanner with the orbito-meatel line parallel to the transaxial plane of the tomograph. Head position was monitored via laser crosshairs and video camera.

### Image quantification

Parametric images of specific FDOPA influx constants (*K_i_* maps) were created at a voxel level for the whole brain using linear graphical analysis (Patlak and Blasberg, 1985) of time activity curves with an occipital cortex (Brown et al., 1979) non-specific reference input function. Qualitative summated ADD images created from the dynamic FDOPA time series by integrating all 26 frames of the dynamic image were also produced and then transformed into standard stereotaxic (Montreal Neurological Institute, MNI) space using an FDOPA template created in-house from a healthy volunteer database. These ADD images contain both tracer delivery and specific uptake information and provide adequate anatomical detail to allow them to be stereotaxically normalized into standard MNI space. Subsequently, the *K_i_* maps were individually normalized to MNI stereotaxic space by applying the transformation parameters defined during the normalization of their respective ADD images. This spatial transformation of parametric images made it possible to perform a region of interest (ROI) analysis as described below.

### Region of interest (ROI) analysis

Standard ROI object maps sampling the ventral and dorsal striatum were defined on the MNI single-subject ROI in stereotaxic space. For our striatal ROIs, the volume was subdivided as follows: all planes containing striatal structures below the anterior commissure-posterior commissure plane were operationally defined as the ventral striatum (VS) ROI, and all planes above the anterior commissure-posterior commissure plane containing striatal structures formed the dorsal striatum (DS) ROI. The standard object map was applied to the transformed *K_i_* maps and values of FDOPA *K_i_* (units: ml • g^−1^ • min^−1^) were obtained for the two striatal ROIs for each individual (McGowan et al., 2004). When performing our ROI analysis a manual correction for head movement was applied as previously described (Whone et al., 2003).

### Statistical analysis

We used Pearson partial correlations to examine the relationships between striatal FDOPA *K*_i_ values and disinhibitory NS traits, controlling for relevant nuisance variables (Spector and Brannick, 2011). Statistical significance was set at a Bonferroni-corrected *P* < 0.0125 (i.e., 0.05/4).

## Results

Mean ± SD scores in our sample for NS2 (Impulsivity) and NS3 (Extravagance) were 3.5 ± 2.5 and 4.3 ± 1.1, respectively. These results are comparable to those obtained in a normative sample of 106 UK men (mean age 31, SD ± 11.5) by Otter et al. (1995) (NS2 mean 3.1, SD ± 2.2; NS3 mean 3.8, SD ± 2.0). HA scores (HA mean 8.1, SD ± 4.7) were somewhat lower than those reported by Otter et al. (HA mean 10.7 ± 6.2), perhaps reflecting self-selection bias in individuals who volunteer for PET scanning (Oswald et al., 2013).

Mean ±SD FDOPA *K_i_* values for the VS and DS ROIs were 0.0131 ± 0.001 and 0.0125 ± 0.002 ml • g^−1^ • min^−1^ respectively.

Since, in adults, NS shows a significant decrease with increasing age (Otter et al., 1995), we controlled for the effects of age when examining the relationship between striatal FDOPA *K_i_* and disinhibitory NS traits (Impulsivity and Extravagance). Furthermore, for the reasons outlined above, we additionally controlled for HA scores.

**Figure 1:**
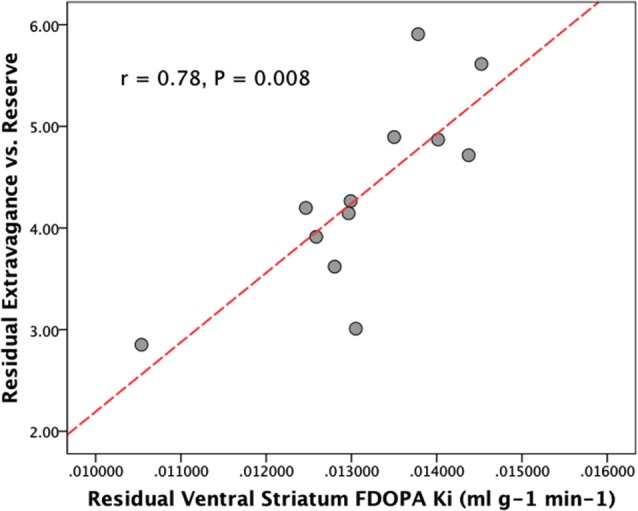
**Plot showing the partial correlation between ventral striatal dopamine synthesis capacity (FDOPA *K_i_*), and disinhibitory personality traits (NS3, Extravagance vs. reserve) controlling for age and Harm Avoidance**.

When controlling for the influence of age and HA there was a significant relationship between VS FDOPA *K_i_* and NS3 (Extravagance) (*r* = 0.78, bootstrap 95% CI 0.52–0.98, *P* = 0.008), but not between VS FDOPA *K_i_* and NS2 (Impulsivity) (*r* = 0.44, *P* = 0.2). There were no significant relations between DS FDOPA *K_i_* and either NS3 (*r* = 0.28, *P* = 0.40) or NS2 (*r* = 0.003, *P* = 0.99) when controlling for age and HA (see Figure 1). Examination of Figure 1 suggests that one individual data point may be an outlier. When this data point was removed, however, the relationship between VS FDOPA *K_i_* and NS3, controlling for age and HA, remained significant (*r* = 0.71, bootstrap 95% CI 0.51–0.92, *P* = 0.014). We found identical results when using a Spearman partial correlation (Schemper, 1991).

## Discussion

Consistent with our hypothesis, we found that, controlling for the effects of age and HA, variation in trait disinhibition was associated with levels of striatal DA synthesis capacity. Individuals with greater levels of trait disinhibition, in particular, tendencies to financial irresponsibility and extravagance, had greater DA synthesis capacity, as indexed by FDOPA *K_i_* values, in the ventral but not dorsal striatum.

We (Lawrence et al., 2013) recently found that individual differences in behavioral disinhibition (using the same personality trait measure as used here) were similarly related to individual differences in ventral striatal DA synthesis capacity in individuals with Parkinson’s disease. Those, individuals, were however, being treated with DA agonist medication, which could potentially have influenced both striatal DA synthesis (Rowlett et al., 1993) and externalizing behaviors, including pathological gambling (Weintraub et al., 2006). The current results importantly extend our earlier findings to healthy, non-medicated individuals, showing a relationship between disinhibitory traits and ventral striatal DA synthesis capacity in the absence of potential DAergic drug-induced effects. Taken together with the finding that genetic variation in DDC activity predicts disinhibitory sensation seeking tendencies in healthy individuals (Derringer et al., 2010), our results suggest that the link between behavioral disinhibition and ventral striatal DA synthesis capacity is likely to be, to a significant extent, genetically mediated. At the same time, we acknowledge that there are substantial (potentially shared) environmental influences on ventral striatal DA synthesis capacity (Stokes et al., 2013), behavioral disinhibition (Lomanowska et al., 2011) and externalizing (Hicks et al., 2013).

As in our earlier study of Parkinson’s disease, here we found that only the r-NS facet-level scale NS3 (Extravagance vs. Reserve) was related to ventral striatal DA synthesis capacity. There was no significant relation with the NS2 subscale (Impulsivity vs. Reflection). The reasons for this are unclear. It is notable, however, that, of the NS facet-level scales, NS3 shows the strongest relation to both pathological gambling (Kim and Grant, 2001; Nordin and Nylander, 2007) and substance abuse (Etter et al., 2003). It may be that, of the disinhibitory NS facets, NS3 most closely indexes those traits (irresponsibility, problematic impulsivity) that lie at the core of the broad externalizing factor (Krueger et al., 2007).

Consistent with the proposal that externalizing vulnerability reflects, at least in part, individual differences in reward sensitivity (Iacono et al., 2008); the influence of variation in ventral striatal DA synthesis capacity on externalizing propensity likely reflects DA’s role in one particular aspect of reward processing, namely the attribution of incentive salience (Berridge, 2012). Incentive salience is a motivational component of reward, one that transforms sensory information about rewards and reward cues into attractive, “wanted” incentives, motivating pursuit (Berridge, 2012). Notably, Flagel et al. (2010) found in rats that incentive salience attribution and behavioral disinhibition are genetically influenced, correlated traits. Available data suggest that animals prone to attribute incentive salience to reward cues have a more active DA system than those who do not (Flagel et al., 2010). In humans, VS FDOPA *K_i_* values have been found to positively correlate with BOLD-fMRI activity to reward cues in limbic brain regions linked to incentive salience attribution (Siessmeier et al., 2006), and limbic BOLD-fMRI responses to reward cues are correlated with both disinhibitory personality traits (Beaver et al., 2006; Buckholtz et al., 2010) and externalizing symptomatology (Bjork et al., 2010). One possibility is that individuals high on externalizing risk show exaggerated phasic DA release to reward cues, resulting from a larger releasable pool of DA generated by increased DA synthesis capacity (Bello et al., 2011; Anzalone et al., 2012), triggering excessive attribution of incentive salience to environmental cues and their associated rewards, leading to behavioral disinhibition (Flagel et al., 2010; Lovic et al., 2011) (but see Huys et al., 2014 for an alternative proposal).

At first glance, our findings seem inconsistent with an earlier study of detoxified alcoholics, which found no differences in ventral striatal DA synthesis capacity relative to a healthy control group (Heinz et al., 2005). Alcohol misuse, however, is multiply determined, and influenced to a greater extent by factors unique to alcohol, than by the general tendency to externalizing (Krueger et al., 2007). Further, it is possible that chronic alcohol use may produce potentially neurotoxic effects on DA neurons (Gilman et al., 1998), obscuring any pre-morbid trait influence on DA synthesis capacity.

It is important to note that FDOPA is not a specific ligand for DA neurons but rather is metabolized by all neurons that contain AADC (Brown et al., 1999). Hence, it is a marker for all tissues that take up and store monoamines, including serotonin (5-hydroxytryptamine, 5-HT) as well as DA neurons (Hashemi et al., 2012). 5-HT has been implicated in various aspects of impulsivity (Carver et al., 2008; Cools et al., 2008). Notably, Jupp et al. (2013) however, failed to find a relationship between levels of trait impulsivity (defined by premature responding on a 5-choice serial reaction time task) and levels of accumbens 5-HT in rats. It is likely, therefore, that individual differences in trait disinhibition are primarily related to individual differences in ventral striatal DA synthesis capacity.

In conclusion, we have found that personality based vulnerability to externalizing problems, including pathological gambling, is related to relatively increased DA synthesis capacity in the ventral, but not dorsal, striatum in a sample of healthy men. Our results are consistent with preclinical models of behavioral disinhibition and addiction proneness, and may prove informative in understanding the neurobiological and psychological mechanisms underlying personality risk for phenotypically diverse forms of disinhibitory psychopathology.

## Conflict of interest statement

The authors declare that the research was conducted in the absence of any commercial or financial relationships that could be construed as a potential conflict of interest.
